# Decrease of vitamin D concentration in patients with HIV infection on a non nucleoside reverse transcriptase inhibitor-containing regimen

**DOI:** 10.1186/1742-6405-7-40

**Published:** 2010-11-23

**Authors:** Anali Conesa-Botella, Eric Florence, Lutgarde Lynen, Robert Colebunders, Joris Menten, Rodrigo Moreno-Reyes

**Affiliations:** 1 Department of Clinical Sciences, Institute of Tropical Medicine, Antwerp, Belgium; 2 Department of Epidemiology and Social Medicine, University of Antwerp, Belgium; 3 Clinical Trials Unit, Institute of Tropical Medicine, Antwerp, Belgium; 4 Department of Nuclear Medicine, Erasmus Hospital, Free University of Brussels, Belgium

## Abstract

**Background:**

Vitamin D is an important determinant of bone health and also plays a major role in the regulation of the immune system. Interestingly, vitamin D status before the start of highly active antiretroviral therapy (HAART) has been recently associated with HIV disease progression and overall mortality in HIV-positive pregnant women. We prospectively studied vitamin D status in HIV individuals on HAART in Belgium.

We selected samples from HIV-positive adults starting HAART with a pre-HAART CD4 T-cell count >100 cells/mm^3 ^followed up for at least 12 months without a treatment change. We compared 25-hydroxyvitamin D plasma [25-(OH)D] concentration in paired samples before and after 12 months of HAART. 25-(OH)D levels are presented using two different cut-offs: <20 ng/ml and <30 ng/ml.

**Results:**

Vitamin D deficiency was common before HAART, the frequency of plasma 25-(OH)D concentrations below 20 ng/ml and 30 below ng/ml was 43.7% and 70.1% respectively. After 12 months on HAART, the frequency increased to 47.1% and 81.6%.

HAART for 12 months was associated with a significant decrease of plasma 25-(OH)D concentration (p = 0.001). Decreasing plasma 25-(OH)D concentration on HAART was associated in the multivariate model with NNRTI-based regimen (p = 0.001) and lower body weight (p = 0.008). Plasma 25-(OH)D concentrations decreased significantly in both nevirapine and efavirenz-containing regimens but not in PI-treated patients.

**Conclusions:**

Vitamin D deficiency is frequent in HIV-positive individuals and NNRTI therapy further decreases 25-(OH)D concentrations. Consequently, vitamin D status need to be checked regularly in all HIV-infected patients and vitamin D supplementation should be given when needed.

## Background

Vitamin D status is an important determinant of bone health. Vitamin D deficiency increases the risk of osteoporosis and fractures and in its most severe form causes rickets in children and osteomalacia in adults. In addition, a large number of studies indicate that vitamin D also plays an important role in insulin secretion, lipid metabolism, autoimmune disorders, cell proliferation, and cardiovascular diseases [1-8].

The main determinant of vitamin D status is the intra-epidermal conversion of pre-vitamin D into vitamin D by ultra violet radiation. As a consequence, increased skin pigmentation and low sunlight exposure are risk factors for vitamin D deficiency [2]. Cholecalciferol is metabolized into 25-dihydroxyvitamin D [25-(OH)D] in the liver and into its active form 1,25-dihydroxyvitamin D [1,25-(OH)_2_D] in the kidney and peripheral cells such as activated immune cells [8] by two successive hydroxylations by cytochromes P450 [9]. Both 1,25-(OH)_2_D and 25-(OH)D are catabolized by the cytochrome CYP24 [9]. In target cells such as immune cells, 1,25-(OH)_2_D is converted locally at the site of action [8]. Due to its longer half-life of 15 days, 25-(OH)D is considered the best marker of vitamin D status [8].

Although there is no agreement among international experts on the most appropriate cut-off value for adequate vitamin D level, individuals with 25-(OH)D below 20 ng/ml are considered as deficient [2]. However, a value above 30 ng/ml has been suggested to be associated in the general population with better health outcomes such as higher bone mineral density, less falls and fractures as well as protection against cancer [10]. Lower vitamin D level has recently been associated with increased mortality in the general population [11] as well as with HIV disease progression and overall mortality in a cohort of Tanzanian pregnant women with HIV infection [12].

Paul et al. showed that vitamin D deficiency was more prevalent among HIV-positive patients treated with HAART when compared to HAART-naive patients and negative controls [13], however, Ramayo et al. showed opposite results [14]. In addition, HAART may impair vitamin D metabolism [15,16] as shown by some *in vitro *studies. Cozzolino et al. [16] demonstrated the inhibition by protease inhibitors (PIs) of the 25-hydroxylase and the 1α-hydroxylase involved in vitamin D metabolism. Ellfolk et al. [17] showed the inhibition of the 25-hydroxylase by efavirenz, a non-nucleoside reverse transcriptase inhibitor (NNRTI).

Vitamin D deficiency and bone disease in HAART patients [14,18,19] have been associated with NNRTIs [20-22], as well as tenofovir [23,24], and PIs [19,24-26]. However, there is a lack of longitudinal studies on vitamin D variation during HAART; therefore we studied vitamin D status in a longitudinal cohort study of AIDS patients during the first year of HAART.

## Methods

### Subjects

Subjects were HIV-positive individuals belonging to the outpatient cohort of the Institute of Tropical Medicine (ITM) in Antwerp, Belgium. Antwerp is situated on the 51^st ^degree of latitude and receives 1000 hours of sun per year, half the exposure seen at the equator.

Using the ITM cohort electronic database we selected retrospectively patients fitting the following criteria: (a) HIV-positive adults starting HAART with CD4 T-cell counts >100 cells/mm^3^; (b) followed up for at least 12 months; (c) without a treatment change in the first year of HAART. Individuals with a CD4 T-cell count below 100 cell/mm^3 ^were excluded as they are often acutely unwell and may have other factors contributing to vitamin D deficiency.

Individuals were started either on a PI-based or on an NNRTI-based regimen. Patients on NNRTI were either on Nevirapine (= 20) or on Efavirenz (n = 23). Sixty-six percent (n = 29) of patients on PI were on a boosted regimen with Ritonavir. Twenty-seven individuals were taking Tenofovir; 18 in combination with NNRTIs and 8 with PIs. Patients taking corticosteroids, suffering from "severe renal disease" or "severe liver disease", or with an active granulomatous disease such as active tuberculosis, sarcoidosis and Crohn's disease were excluded. "Severe renal disease" was defined as urea and creatinine twofold above the normal reference values. "Severe liver disease" was defined as a patient having both elevated alanine aminotransferase and aspartate aminotransferase 5 times above the normal reference values.

All subjects had signed an informed consent allowing additional investigation for research purpose on the stored plasma samples left over of routine blood testing. The study was approved by the institutional review board of the ITM.

### Study design

We selected individuals who had stored samples available at the start of HAART and 12 months later. We compared paired pre-HAART and post-HAART samples. The pre-HAART sample was drawn between the start of HAART and maximum 3 months before it. The 12 months sample was drawn minimum 3 months before and maximum 3 months after 12 months of HAART.

### Clinical data

Clinical data were extracted and analyzed anonymously. We recorded the following variables: sex, skin color, season, age, weight, CD4 T-cell nadir and pre-HAART CD4 T-cell level, viral load, HIV disease stage, HAART regimen, total cholesterol, HDL-C, and LDL-C. No data on non prescribed vitamin D supplementation or sun exposure were routinely collected.

### Laboratory analysis

Plasma samples were selected among stored samples obtained between 1997 and 2009. Plasma had been isolated by centrifugation of blood drawn on ethylenediaminetetraacetic acid (EDTA)-containing tubes and aliquots had been kept in a -80°C freezer.

Total plasma 25-(OH)D was measured by radioimmunoassay (DiaSorin). The interassay coefficient of variation was 9-13%. The quality control was performed by the vitamin D External Quality Assurance Survey (DEQAS). All samples were measured in duplicate. CD4 T-cell count was determined by standard flow cytometry (FACScalibur, Becton Dickinson). Viral load was measured with the Cobas Amplicor HIV-1 (Roche). Total cholesterol, HDL-C, and LDL level were determined by automated standard laboratory techniques.

### Vitamin D status

Plasma 25-(OH)D concentrations are presented using two different cut-offs, <20 ng/ml (50 nmol/l) and <30 ng/ml (75 nmol/l).

### Statistical analysis

All statistical analyses were performed with STATA and R software. Pre-HAART data was summarized using counts and percentages, means and standard deviations for normally distributed data, and median (interquartile range, IQR) for non-normal continuous variables. The difference between PI and NNRTI groups was assessed by Fisher's exact test for percentages, t-test for means and Mann-Whitney for medians. Normality was assessed using graphical methods and confirmed by D'agostino and Pearson omnibus normality test. Within-group or overall changes in vitamin D levels were assessed using a paired t-test. Logistic regression or linear regression methods were used to study the relation between the predictors and vitamin D. A threshold of p < 0,1 was used for variable inclusion in the multivariate model, which was then simplified by backward elimination. P-values presented in the text refer to the final statistical model obtained.

## Results

### Subject characteristics

Among the 194 patients fitting the inclusion criteria, plasma samples were available at both pre-HAART and 12 months for 89 patients. Two patients were excluded because of chronic liver disease. None of the patients had renal insufficiency, a known granulomatous disease or were treated with corticosteroids during the study period. A mean ± SD of 381 ± 39 days was observed between both time points.

The pre-HAART population characteristics are summarized in Table 1. No significant differences at baseline were observed between patients on PI and NNRTI-based regimen. The "dark skin" group included 17 individuals from Central and Southern Africa; the 'light skin" group included 68 Caucasians, one Moroccan and one Ecuadorian.

**Table 1:** Pre-HAART characteristics of the study population.

Variables		NNRTI	PI	p value
		n = 43	N = 44	
25-(OH)D (ng/ml)	mean ± SD	26.6 ± 13.5	22.6 ± 8.9	0.101
Sex Male	n (%)	38 (88.4)	34 (77.3)	0.256
Dark skin color	n (%)	7 (16.3)	10 (22.7)	0.590
Pre-HAART sampling				
Winter	n (%)	15 (34.9)	17 (38.6)	0.657
Spring	n (%)	5 (11.6)	8 (18.2)	
Summer	n (%)	11 (25.6)	11 (25.0)	
Fall	n (%)	12 (27.9)	8 (18.2)	
Age (years)	median (IQR)	38.6 (30.8;44.8)	37 (31.8;44.6)	0.929
Weight (Kg)^α^	median (IQR)	73 (68;85)	70.5 (65.3;83.5)	0.675
CD4 (cells/mm^3^)				
Nadir	median (IQR)	224 (181;292)	247.5 (182;314.5)	0.61
pre-HAART	median (IQR)	254 (183;338)	299 (241;390.5)	0.052
Viral Load (log_10_)	median (IQR)	5.24 (4.95;5.53)	5.5 (4.97;5.86)	0.168
HIV stage (CDC)				
A	n (%)	31 (72.1)	32 (72.7)	0.640
B	n (%)	8 (18.6)	6 (13.6)	
C	n (%)	4 (9.3)	6 (13.6)	

PI: Protease inhibitors; NNRTI: non-nucleoside reverse transcriptase inhibitors; SD: Standard deviation; IQR: Interquartile range; ^α ^n = 85

### Risk of vitamin D deficiency in HIV individuals before HAART

Before HAART, 43.7% and 70.1% of the individuals had plasma 25-(OH)D concentrations below 20 ng/ml and 30 ng/ml, respectively. In multivariate analysis, dark-skinned individuals had 8.9 and 11.2 times more risk than light-skinned individuals to present with plasma 25-(OH)D concentrations below 20 ng/ml and 30 ng/ml (p = 0,001 and 0,026), respectively. In addition, the prevalence of plasma 25-(OH)D concentrations below 30 ng/ml was higher in winter compared to summer (p = 0.001) and fall (p = 0.020). There was no significant effect of gender, age, weight or HIV disease stage, pre-HAART CD4 T-cell count or CD4 T-cell nadir, or viral load on vitamin D levels.

### Risk of vitamin D deficiency of HIV individuals during HAART

After 12 months on HAART, 47.1% and 81.6% of individuals has plasma 25-(OH)D concentrations below 20 ng/ml and 30 ng/ml respectively. Individuals with a darker skin color or being treated by a NNRTI-based regimen presented an increased risk of having plasma 25-(OH)D concentrations lower than 20 ng/ml (respectively 6 and 3 fold; p = 0,006 and p = 0,020). Individuals with a low body weight were 4.7 times more at a risk of having plasma 25-(OH)D concentrations below 30 ng/ml (p = 0,026). There was no influence of sex, sampling season, pre-HAART CD4-T cell count and CD4 T-cell count nadir, viral load and HIV stage.

**Figure 1:**
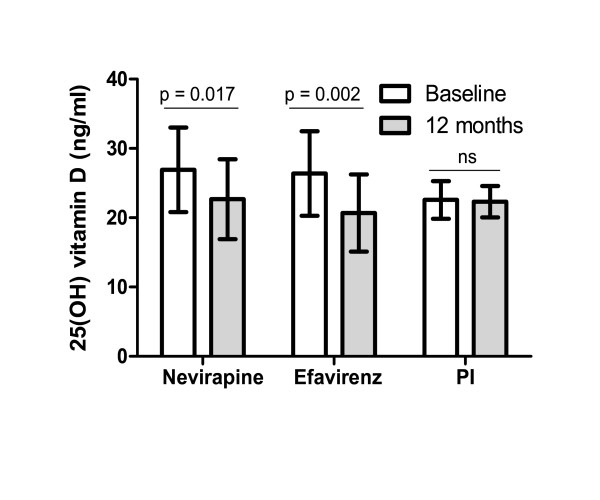
**plasma 25(OH)D concentration pre-HAART and after 12 months in individuals on NNRTI-based regimen (Nevirapine and Efavirenz) and PI-based regimen**. PI: protease inhibitor.

When analyzing paired pre-HAART and post-HAART samples from individuals on HAART for 12 months, we observed a significant decrease of plasma 25-(OH)D concentration in the studied population (Table 2). The decrease of 25-(OH)D was associated in the multivariate model to NNRTI-based regimen (p = 0.001) and to a lower body weight (p = 0.008). Moreover, plasma 25-(OH)D concentration decreased significantly after 12 months on NNRTI regimen (both on nevirapine and on efavirenz), but not in PI-treated patients (Figure 1).There was no association between the use of tenofovir and a decrease in vitamin D levels (p = 0.665; data not shown).

**Table 2:** Determinants of 25(OH)D (ng/ml) decrease on HAART

Variable	25(OH)D (CI) pre-HAART	25(OH)D (CI) post-HAART	Adj p
Population	24.6 (22.1;27.0)	22.0 (19.8;24.1)	0.001*
Sex			0.668
M	25.6 (22.8;28.4)	22.8 (20.4;25.3)	
F	19.6 (14.8;24.4)	17.8 (14.0;21.6)	
Skin color			0.195
Light	26.7 (24.0;29.4)	23.6 (21.2;26.0)	
Dark	15.8 (11.9;19.7)	15.3 (11.6;19.0)	
Pre-HAART sampling			0.127
Winter	21.9 (18.1;25.6)	19.8 (16.2;23.5)	
Spring	22.1 (16.5;27.6)	22.4 (19.3;25.5)	
Summer	29.8 (24.2;35.4)	22.4 (19.7;29.0)	
Fall	24.8 (19.1;30.5)	22.5 (16.8;28.2)	
Age			0.644
<35 years	24.9 (21.0;28.7)	22.2 (18.6;25.8)	
35-50 years	24.2 (20.2;28.2)	21.2 (17.8;24.5)	
>50 years	24.8 (20.0;29.6)	24.2 (19.2;29.2)	
Weight (Kg)			0.008
≤70	23.0 (19.4;26.6)	18.5 (16.2;20.9)	
>70	25.8 (22.4;29.2)	24.8 (21.4;28.1)	
CD4 (CD4/mm^3^) nadir			0.160
≤200	25.4 (21.0;29.8)	21.3 (18.2;24.5)	
>200	24.1 (21.1;27.1)	22.3 (19.4;25.3)	
CD4 (CD4/mm^3^) pre-HAART			0.174
≤200	25.9 (20.5;31.4)	21.5 (17.4;25.6)	
>200	24.1 (21.3;26.9)	22.1 (19.5;24.8)	
Viral Load (log_10_)			0.666
>5	25.7 (22.9;28.6)	22.9 (20.3;25.6)	
≤5	21.7 (16.7;26.6)	19.6 (15.8;23.5)	
HIV stage (CDC)			0.760
A	25.4 (22.4;28.4)	22.7 (20.0;25.3)	
B	24.3 (18.0;30.5)	21.0 (15.7;26.4)	
C	19.8 (13.0;26.5)	18.7 (11.8;25.6)	
Therapy			0.001
NNRTI	26.6 (22.5;30.8)	21.6 (17.8;25.5)	
PI	22.6 (19.9;25.3)	22.3 (20.1;24.6)	

p: p-value for comparison between groups (linear regression); adj p: p-value from final multivariate model (after backwards elimination); CI: 95% confidence interval; * non adjusted p value

### Changes in cholesterol HDL-C and LDL-C levels on HAART

After one year of HAART, we observed a significant increase in cholesterol concentration (from 168.5 to 202.5 mg/dl; p < 0.001; data not shown) and HDL-C levels (from 40.8 and 46.6 mg/dl; p = 0.002; data not shown). LDL-C level did not increase significantly (p = 0.168; data not shown). There was no association between the increase of lipids and the pre-HAART vitamin D status or with the type of HAART.

## Discussion

Our study shows a high prevalence of vitamin D deficiency in HIV-infected individuals as observed by others [13,27,28]. As reported in the general population [2,29], vitamin D deficiency during summer and fall was lower than during winter.

We also showed that a NNRTI-based treatment was associated with a significant decrease of 25(OH)D plasma concentration after 12 months on HAART. Similarly to our findings, Van den Bout-van den Beukel [21] described a prevalence of vitamin D deficiency of 62% in dark-skinned individuals in a cross-sectional study of HIV-positive individuals. In their study, patients on an NNRTI-based therapy had lower vitamin D levels than those on a PI-based therapy.

Decreased bone mineral density is often described in cross sectional studies in patients on tenofovir (NRTI) [23,30] or PIs [19,24-26]. However, we and others found an association between NNRTI-based regimen and vitamin D levels [21,22]. The lack of concordant findings on the effect of HAART on bone mineral density and vitamin D might result from the direct action of antiretroviral drugs on osteoclasts and osteoblasts [26,31,32]. The effect of NRTI drugs on vitamin D metabolism has never been studied but interactions are unlikely as NRTIs are not metabolized by cytochromes [33]. In contrast, PI and NNRTI drugs have been shown to interfere with cytochromes involved in the vitamin D metabolism (e.g. NNRTI induce CYP3A4; PIs inhibit CYP3A4) [9,16,17,33-35]. The role of each antiretroviral drug particularly from the NNRTI family on vitamin D metabolism should be evaluated further to better understand the action of HAART on enzymes involved in vitamin D metabolism.

Our data show that patients with a body weight ≤ 70 kg had a higher risk of plasma 25-(OH)D concentration below 30 ng/ml after one year on HAART. A similar association has been reported, in HIV-infected individuals with BMI and vitamin D status [36]. These results are different from those of non HIV-infected patients, where obesity is associated with lower plasma 25-(OH)D concentrations [37] and a slower increase of vitamin D concentration in response to UVB irradiation. In non-HIV infected individuals, this has been explained by an altered release of vitamin D from the skin into the circulation, and the decreased bioavailability of vitamin D by deposition in body fat compartment [37].

This study has several limitations. The small sample size precluded determination of whether the decrease of plasma 25-(OH)D concentration was associated with the use of a certain HAART combination. In addition, the retrospective design and the lack of detailed information about UV radiation exposure and over the counter multivitamin supplementation limited our study.

Our results suggest that HIV patients and particularly those treated with HAART represent a population with higher risk of vitamin D deficiency. Beyond bone health, vitamin D deficiency is associated with many chronic diseases such as cancer, cardiovascular disease, diabetes and immunological diseases including HIV as well as chronic pain in HIV-infected individuals [10,11,38-41].

Vitamin D supplementation to maintain an optimal plasma 25-(OH)D concentration above 30 ng/ml is an inexpensive and a safe measure as vitamin D toxicity is only observed at plasma 25-(OH)D concentration higher than 150 ng/ml [2]. Therefore, clinicians taking care of HIV patients should be aware of the risk of vitamin D deficiency associated with HIV and HAART and the benefits of its supplementation.

## Conclusions

In conclusion, our findings suggest that vitamin D deficiency is highly prevalent in HIV individuals and that NNRTI therapy further decreases 25-(OH)D concentrations. Consequently, vitamin D status need to be checked regularly in all HIV-infected patients and vitamin D supplementation should be given when needed.

## Competing interests

The authors declare that they have no competing interests.

## Authors' contributions

CBA extracted the data, performed the data analysis, and wrote the paper. FE, LL, MRR, and CR. collaborated in conceiving the study and in the writing and reviewing of the article MJ collaborated to the statistical analysis. All authors read and approved the final manuscript.
